# Combining biomarkers to construct a novel predictive model for predicting preoperative lymph node metastasis in early gastric cancer

**DOI:** 10.3389/fonc.2025.1533889

**Published:** 2025-05-08

**Authors:** Yujian He, Xiaoli Xie, Bingxue Yang, Xiaoxu Jin, Zhijie Feng

**Affiliations:** Department of Gastroenterology, The Second Hospital of Hebei Medical University, Hebei Key Laboratory of Gastroenterology, Hebei Institute of Gastroenterology, Hebei Clinical Research Center for Digestive Diseases, Shijiazhuang, Hebei, China

**Keywords:** early gastric cancer, lymph node metastasis, nomogram, *HAVCR1*, predictive model

## Abstract

**Background:**

Accurately identifying the status of lymph node metastasis (LNM) is crucial for determining the appropriate treatment strategy for early gastric cancer (EGC) patients.

**Methods:**

Univariate and multivariate logistic regression analyses were used to explore the association between clinicopathological factors and LNM in EGC patients, leading to the development of a nomogram. Differential expression analysis was conducted to identify biomarkers associated with LNM, and their expression was evaluated through immunohistochemistry. The biomarker was integrated into the conventional model to create a new model, which was then assessed for reclassification and discrimination abilities.

**Results:**

Multivariate logistic regression analysis revealed that tumor size, histological type, and the presence of ulcers are independent risk factors for LNM in EGC patients. The nomogram demonstrated good clinical performance. Incorporating *HAVCR1* immunohistochemical expression into the new model further improved its performance, reclassification, and discrimination abilities.

**Conclusion:**

The novel nomogram predictive model, based on preoperative clinicopathological factors such as tumor size, histological type, presence of ulcers, and *HAVCR1* expression, provides valuable guidance for selecting treatment strategies for EGC patients.

## 1 Introduction

Gastric cancer (GC) is the fifth most common cancer and the fourth leading cause of cancer-related death worldwide (1). In China, it ranks third in both incidence and mortality among all cancer types (2). Early gastric cancer (EGC) is defined as a tumor confined to the mucosal or submucosal layers of the stomach, regardless of lymph node metastasis (LNM) (3). LNM is recognized as one of the most critical prognostic factors in EGC (4). The presence of LNM not only profoundly influences the overall survival and recurrence rates of EGC patients but also plays a crucial role in determining the appropriate treatment strategies (5). For EGC patients without LNM who are suitable for surgery, minimally invasive procedures like endoscopic mucosal resection (EMR) and endoscopic submucosal dissection (ESD) are commonly used (6). For those at risk of LNM, standard gastrectomy with D2 lymph node dissection is the preferred approach (7). Therefore, accurately predicting the status of LNM in EGC before surgery is of great clinical importance.

However, accurately assessing the risk of LNM before surgery remains a significant clinical challenge. Currently, the National Comprehensive Cancer Network (NCCN) has not recommended any specific imaging modality for the accurate detection of LNM (8). Although modalities such as multidetector computed tomography (MDCT), endoscopic ultrasonography (EUS), magnetic resonance imaging (MRI), and positron emission tomography-computed tomography (PET-CT) are available, none of these methods can reliably and accurately evaluate the lymph node status in gastric cancer patients (9, 10). Although many studies have developed models to predict LNM, most of these models incorporate risk factors that can only be determined postoperatively, such as tumor invasion depth, presence of lymphatic invasion, vascular invasion, and pathological T staging (11–18). These indicators are not available preoperatively. And Some researchers have utilized multiphoton imaging to extract and analyze collagen characteristics in tumor specimens to predict LNM. However, this approach involves highly complex techniques and is associated with significant costs, limiting its widespread application (19, 20). In recent years, studies have developed sensitive biomarkers for predicting LNM through transcriptomic analysis. These studies have established predictive models composed of multiple genes or long non-coding RNA (LncRNA) combined with clinicopathological factors to enhance the identification of LNM (21, 22). However, these studies require the expression analysis of multiple genes in patient tissues, which limits their clinical applicability.

To the best of our knowledge, no studies have yet utilized transcriptomic analysis to identify biomarkers for immunohistochemical (IHC) analysis of protein expression in preoperative biopsy tissues, combined with preoperative clinicopathological factors, to develop a model for predicting LNM in EGC. Therefore, this study aims to enhance the predictive performance of a conventional model developed using preoperative clinicopathological factors by incorporating IHC expression results of identified biomarkers. This improved model will provide critical guidance for selecting treatment strategies and evaluating prognosis in patients with EGC.

## 2 Materials and methods

### 2.1 Patient recruitment

**Figure 1:**
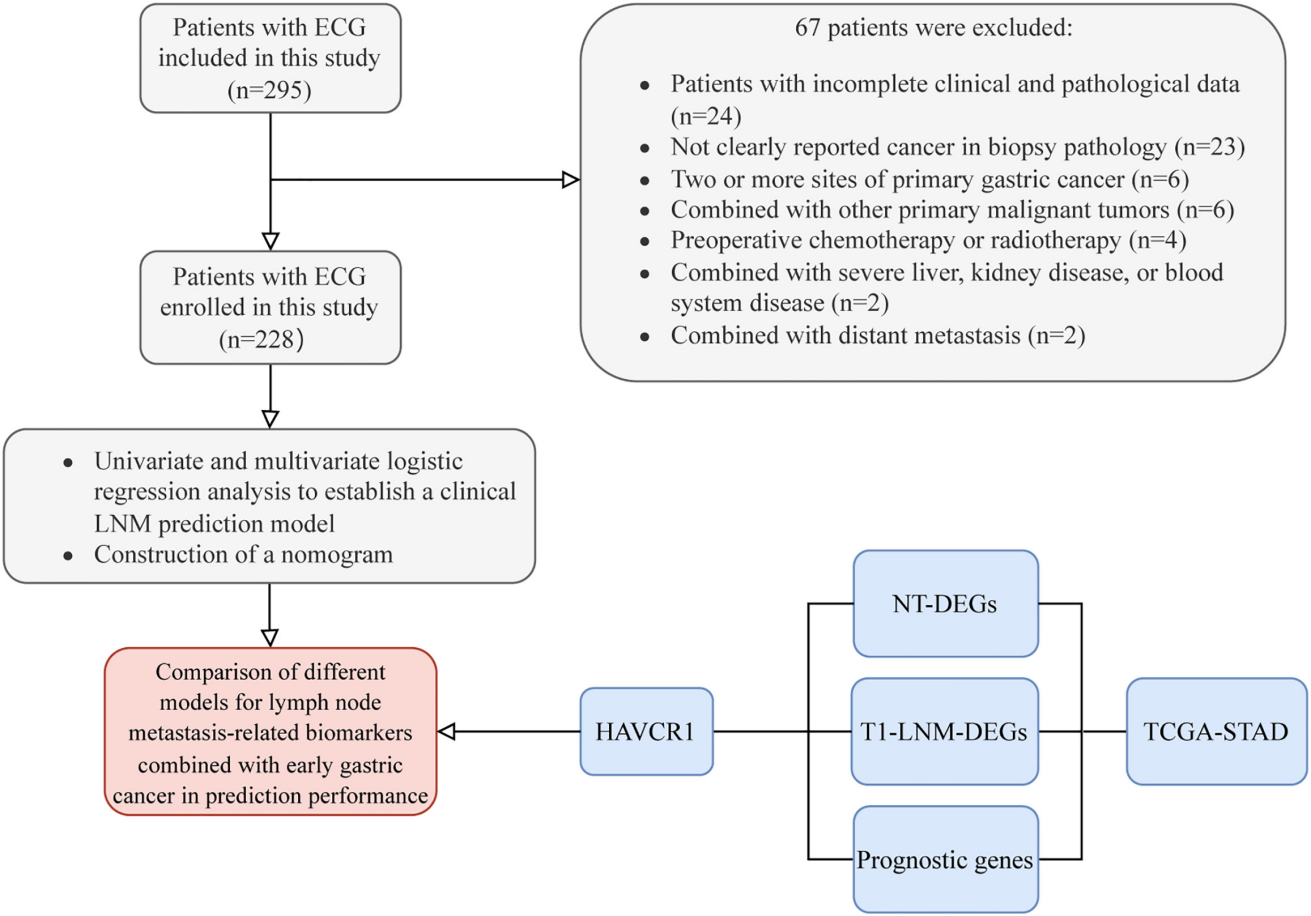
Flow diagram of the study design. EGC, early gastric cancer; LNM, lymph node metastasis; NT, Normal-vs-Tumor; DEGs, differentially expressed genes.

We conducted a retrospective analysis of patients who underwent early gastric cancer resection at the Second Hospital of Hebei Medical University between November 2009 and April 2024 (**Figure 1**). Out of 295 patients reviewed, 228 met the inclusion criteria. Eligible patients were those undergoing their first curative gastrectomy with standard D1+/D2 lymph node dissection and were histopathologically confirmed to have early-stage gastric cancer. They also underwent comprehensive preoperative assessments including blood tests, gastroscopy with biopsy, and CT scans. Sixty-seven patients were excluded based on the following criteria: 1) incomplete clinical pathological data; 2) preoperative biopsy not confirming cancer or without a clear cancer report; 3) patients with multiple primary gastric cancer sites; 4) patients with other primary malignancies; 5) patients who had undergone chemotherapy or radiotherapy prior to surgery; 6) patients with severe liver, kidney, or hematologic diseases; 7) patients with distant metastases. It is worth noting that EGC patients who received minimally invasive treatments, such as ESD or EMR, were excluded because, without standard lymph node dissection, it is not possible to accurately determine whether LNM is present. Based on the presence or absence of LNM in surgical pathology specimens, patients were categorized into two groups: those with LNM and those without LNM. This study was approved by the Ethics Committee of the Second Hospital of Hebei Medical University (2024-R532), conducted in accordance with the ethical standards of the Helsinki Declaration, and informed consent was obtained from all participants.

### 2.2 Clinical pathological data collection

Characteristics of patients, including age and gender, were collected along with data from electronic medical records, endoscopy information platforms, and pathology databases. Information gathered from electronic medical records included family history of gastrointestinal cancer, alcohol consumption history, smoking history, and CT imaging data. Laboratory parameters such as fecal occult blood test (FOBT), albumin (ALB), fibrinogen (Fib), lactate dehydrogenase (LDH), white blood cells (WBC) count, neutrophils (NE) count, lymphocytes (LY) count, monocytes (MO) count, hemoglobin (HGB), neutrophil-to-lymphocyte ratio (NLR), and monocyte-to-lymphocyte ratio (MLR) were also gathered. CT scans assessed for enlarged lymph nodes were confirmed by two independent radiologists. Preoperative gastroscopy data from the endoscopy platform included lesion location, tumor size, endoscopic macroscopic features, the presence of ulcers, background of atrophy/intestinal metaplasia, spontaneous bleeding, lesion color, and clarity of boundaries. Lesion sites were categorized as cardia/fundus, gastric body, or antrum/pylorus. Tumor size was recorded as the maximum diameter measured during gastroscopy. Macroscopic features were classified according to the Paris classification (23) into protruded, superficial, or excavated types. Pathology results obtained from the pathology platform divided pathological types into differentiated (highly or moderately differentiated tubular and papillary adenocarcinomas) and undifferentiated (poorly differentiated adenocarcinoma, signet ring cell carcinoma, and mucinous carcinoma).

### 2.3 Identification of biomarkers associated with lymph node metastasis in early gastric cancer

The RNA sequencing (RNA-seq) data was downloaded from The Cancer Genome Atlas (TCGA) public data platform (https://portal.gdc.cancer.gov/) which included samples from 412 stomach adenocarcinoma (STAD) tissues and 36 normal tissues. Pathological stage T1 RNA-seq data, including 16 LNM-negative and 5 LNM-positive samples, were selected for further analysis. Differential gene expression analysis was conducted using the “edgeR” package (24) comparing tumor versus normal samples and T1 stage LNM versus non-LNM samples. Significant thresholds were set at a false discovery rate (FDR) < 0.05 and |log2 Fold Change (FC)| > 4.0 and |log2 FC| > 1.0, respectively. Genes identified at the intersection of significant differential expression and prognostic relevance were considered potential biomarkers for LNM, visualized in Venn diagrams using the “ggVennDiagram” package (25). The “ggplot2” package was used to create volcano plots. Univariate Cox analysis with the “survival” package distinguished genes with prognostic value, and visualize survival curves using the “survminer” package.

### 2.4 Immunohistochemistry

Paraffin-embedded tissue sections were deparaffinized with xylene and rehydrated through a graded ethanol series. Antigens were retrieved by high-pressure heat treatment, followed by cooling to room temperature. Endogenous peroxidase was blocked using 3% H2O2 for 10 minutes. The sections were then incubated overnight at 4°C with the primary antibody Hepatitis A Virus Cellular Receptor 1 (*HAVCR1*) (final dilution 1:100; SAB; #31156). Afterward, a universal Horseradish Peroxidase (HRP) -conjugated enhanced enzyme-labeled goat anti-rabbit secondary antibody (ZSGB-BIO; PV-9001) was applied and incubated at 37°C for 30 minutes. The staining was developed with diaminobenzidine (DAB), counterstained with hematoxylin, dehydrated, and coverslipped.

### 2.5 Evaluation of immunohistochemical staining

All stained slides were independently evaluated by two experienced researchers who were blinded to the clinical pathological data. The IHC score for *HAVCR1* expression was determined by multiplying the staining intensity by the percentage of positive cells in the tissue sections (26). Staining intensity was scored as 0 (negative), 1 (weak), 2 (moderate), and 3 (strong). The average percentage of marker-positive tumor cells in a given sample was calculated across five regions at 400x magnification. The proportion of positive cells was categorized as follows: 0 (0%-5%), 1 (6%-25%), 2 (26%-50%), 3 (51%-75%), and 4 (76%-100%). The final *HAVCR1* expression score ranged from 0 to 12. For further analysis, all EGC patients were divided into low and high *HAVCR1* expression groups based on the median IHC score of 3.5.

### 2.6 Statistical analysis

Data analysis and graphing were performed using GraphPad Prism (Version 9.5), IBM SPSS Statistics (Version 25.0), and R software (Version 4.3.2). Normality tests were conducted for continuous variables. For normally distributed variables, Student’s t-test was used, while the Mann-Whitney U test was applied for non-normally distributed data. Categorical variables were compared using the chi-square test or Fisher’s exact test, as appropriate. Binary logistic regression was employed to analyze risk factors for LNM. Variables with significant differences (*P*-value < 0.01) in univariate analysis were included in the multivariate logistic regression analysis to identify independent risk factors. In the multivariate analysis, variables with a *P*-value < 0.05 were considered independent risk factors. These factors were then used to construct a predictive model and develop a nomogram. Additionally, receiver operating characteristic (ROC) curves were plotted to calculate the area under the curve (AUC) to quantify the nomogram’s discriminative ability. The model’s predictive accuracy was evaluated using the concordance statistic (C-statistic) and calibration curves to assess the agreement between predicted and observed LNM probabilities. Decision curve analysis (DCA) was performed to determine net benefit. Internal validation of the model’s predictive accuracy was conducted using 1,000 bootstrap resamples. Additionally, the net reclassification improvement (NRI) (27) and integrated discrimination improvement (IDI) were calculated to assess the incremental predictive value of biomarkers for LNM risk. The following R packages were used: “rms” for logistic regression, nomogram construction, and calibration curves; “pROC” for ROC curve plotting; “rmda” for DCA analysis; “PredictABEL” for NRI and IDI analysis; and “caret” for Bootstrap internal validation. The corresponding R code, which details the analysis pipeline, has been uploaded to GitHub (URL: https://github.com/liuziyang-1/Logistic). For all comparisons, a two-sided *P*-value < 0.05 was considered statistically significant.

## 3 Results

### 3.1 Baseline characteristics of patients

A total of 228 patients were included in the study based on the inclusion and exclusion criteria. The baseline characteristics are summarized in **Table 1**. Among the 228 patients, 21 were confirmed to have LNM, resulting in a metastasis rate of 9.2%. Of the patients, 119 (52.2%) had tumors confined to the mucosal layer (T1a), while 109 (47.8%) had tumors invading the submucosal layer (T1b), with LNM positivity rates of 5.9% and 12.8%, respectively.

**Table 1:** Characteristics of patients with early gastric cancer.

Variables	Overall (n= 228)	Non-LMN (n=207)	LMN (n=21)	P-value
Gender				0.698
Female	52 (22.8%)	46 (22.2%)	6 (28.6%)	
Male	176 (77.2%)	161 (77.8%)	15 (71.4%)	
Age (years)				**0.037**
≤50	25 (11.0%)	20 (9.7%)	5 (23.8%)	
50-60	62 (27.2%)	54 (26.1%)	8 (38.1%)	
>60	141 (61.8%)	133 (64.3%)	8 (38.1%)	
Family history of gastrointestinal cancer				0.569
Absent	199 (87.3%)	182 (87.9%)	17 (81.0%)	
Present	29 (12.7%)	25 (12.1%)	4 (19.0%)	
Smoking				0.622
Absent	131 (57.5%)	120 (58.0%)	11 (52.4%)	
Present	97 (42.5%)	87 (42.0%)	10 (47.6%)	
Alcoholism				0.972
Absent	173 (75.9%)	157 (75.8%)	16 (76.2%)	
Present	55 (24.1%)	50 (24.2%)	5 (23.8%)	
FOBT				0.379
Negative	194 (85.1%)	178 (86.0%)	16 (76.2%)	
Positive	34 (14.9%)	29 (14.0%)	5 (23.8%)	
Histologic type				**0.001**
Differentiated	150 (65.8%)	143 (69.1%)	7 (33.3%)	
Undifferentiated	78 (34.2%)	64 (30.9%)	14 (66.7%)	
CT-reported LN status				0.088
Absent	204 (89.5%)	188 (90.8%)	16 (76.2%)	
Present	24 (10.5%)	19 (9.2%)	5 (23.8%)	
Tumor location				0.080
Cardia/fundus	81 (35.5%)	78 (37.7%)	3 (14.3%)	
Gastric body	33 (14.5%)	30 (14.5%)	3 (14.3%)	
Antrum/pylorus	114 (50.0%)	99 (47.8%)	15 (71.4%)	
Tumor size (cm)				**<0.001**
≤2	148 (64.9%)	143 (69.1%)	5 (23.8%)	
2-3	50 (21.9%)	41 (19.8%)	9 (42.9%)	
>3	30 (13.2%)	23 (11.1%)	7 (33.3%)	
Macroscopic type				**0.001**
Protruded	23 (10.1%)	22 (10.6%)	1 (4.8%)	
Superficial	170 (74.6%)	160 (77.3%)	10 (47.6%)	
Excavated	35 (15.4%)	25 (12.1%)	10 (47.6%)	
Ulceration				**0.003**
Absent	91 (39.9%)	89 (43.0%)	2 (9.5%)	
Present	137 (60.1%)	118 (57.0%)	19 (90.5%)	
Atrophic/Intestinal metaplasia background				0.901
Absent	144 (63.2%)	131 (63.3%)	13 (61.9%)	
Present	84 (36.8%)	76 (36.7%)	8 (38.1%)	
Spontaneous bleeding				0.240
Absent	159 (69.7%)	142 (68.6%)	17 (81.0%)	
Present	69 (30.3%)	65 (31.4%)	4 (19.0%)	
The color of the lesion				1.000
Red	190 (83.3%)	172 (83.1%)	18 (85.7%)	
White	38 (16.7%)	35 (16.9%)	3 (14.3%)	
Clear boundaries				1.000
Absent	45 (19.7%)	41 (19.8%)	4 (19.0%)	
Present	183 (80.3%)	166 (80.2%)	17 (81.0%)	
ALB (g/L)	41.45 [39.08, 43.80]	41.40 [39.05, 43.80]	41.80 [39.10, 42.60]	0.979
Fib (g/L)	2.92 [2.58, 3.25]	2.94 [2.55, 3.24]	2.90 [2.73, 3.27]	0.599
LHD (mmol/L)	166.00 [148.00, 191.00]	165.00 [147.00, 190.50]	178.00 [155.00, 193.00]	0.192
WBC (10^9^/L)	5.55 [4.60, 6.75]	5.60 [4.60, 6.77]	5.30 [4.50, 6.40]	0.594
NE (10^9^/L)	3.17 [2.50, 4.16]	3.16 [2.50, 4.20]	3.20 [2.20, 3.60]	0.541
LY (10^9^/L)	1.60 [1.26, 2.07]	1.60 [1.25, 2.08]	1.60 [1.30, 2.04]	0.892
MO (10^9^/L)	0.38 [0.30, 0.50]	0.38 [0.30, 0.50]	0.40 [0.34, 0.40]	0.551
HGB (g/L)	137.00 [125.00, 147.00]	137.00 [125.00, 146.50]	130.00 [121.00, 149.00]	0.858
NLR	1.89 [1.38, 2.78]	1.89 [1.38, 2.78]	1.86 [1.23, 2.29]	0.483
MLR	0.23 [0.17, 0.33]	0.23 [0.17, 0.31]	0.23 [0.19, 0.36]	0.434

LNM, lymph node metastasis; FOBT, fecal occult blood test; LN, lymph node; ALB, albumin; Fib, fibrinogen; LHD, lactate dehydrogenase; WBC, white blood cells; NE, neutrophils; LY, lymphocytes; MO, monocytes; HGB, hemoglobin; NLR, neutrophil-to-lymphocyte ratio; MLR, monocyte-to-lymphocyte ratio. Bold values indicate statistically significant results (P < 0.05).

### 3.2 Univariate and multivariate logistic regression analysis

**Figure 2:**
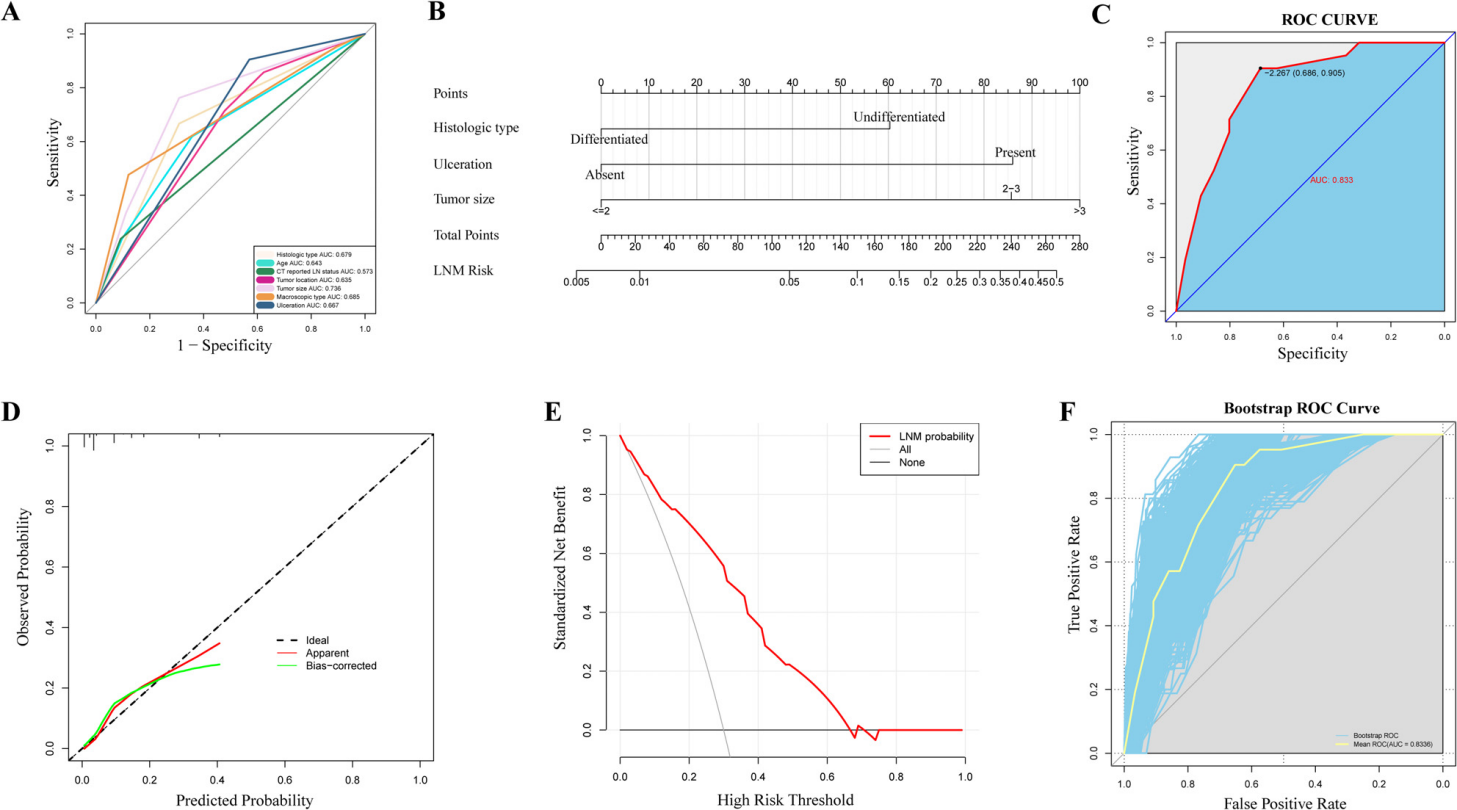
Construction and evaluation of a nomogram for predicting lymph node metastasis. **(A)** Receiver operating characteristic curves of potential risk factors of lymph node metastasis in early gastric cancer by univariate analysis, including histologic type, age, CT reported lymph node status, tumor location, tumor size, macroscopic type and ulceration. **(B)** Nomogram for the prediction of lymph node metastasis in early gastric cancer. **(C)** Receiver operating characteristic curves for the prediction of lymph node metastasis in the early gastric cancer set. **(D)** Calibration curves in the early gastric cancer set. The x-axis represents the predicted probability from the nomogram, and the y-axis is the observed probability of lymph node metastasis in early gastric cancer patients. **(E)** Decision curve analysis demonstrating the net clinical benefit of our predictive model in the early gastric cancer set. The x-axis calculates the threshold probability. The y-axis, standardized net benefit, represents the clinical net benefit after accounting for both the true positive rate and the consequences of false positives. **(F)** The receiver operating characteristic curve was obtained after 1000 Bootstrap validations. The yellow curve represents the mean ROC curve obtained from the bootstrap resamples, highlighting the modelig overall predictive performance. The blue curves represent the individual ROC curves from each of the 1000 resamples, showing the variability of the model’s performance across different data subsets. LN, lymph node; AUC, area under the curve; LNM, lymph node metastasis; ROC, receiver operating characteristic.

We summarized the results of univariate and multivariate logistic regression analyses for factors associated with LNM (**Table 2**). Univariate analysis identified factors such as age, histologic type, CT findings, tumor location, tumor size, macroscopic type, and presence of ulceration as being related to LNM in EGC. These factors were visualized in a univariate ROC curve (**Figure 2A**). Due to the relatively small sample size in this study, we strictly selected variables for modeling by including only those with a p-value < 0.01 in the multivariate analysis. The multivariate analysis revealed that histologic type (Odds Ratio (OR) = 3.12, 95% Confidence Interval (CI): 1.16–9.02, *P* = 0.028), tumor size (OR = 5.03, 95% CI: 1.57–17.80, *P* = 0.008; OR = 6.59, 95% CI: 1.85–25.00, *P* = 0.004), and presence of ulceration (OR = 5.06, 95% CI: 1.33–33.20, *P* = 0.038) were independent risk factors for LNM in EGC patients.

**Table 2:** Univariate and multivariate analysis of preoperative clinicopathological features.

Clinicopathological features	Univariate analysis		Multivariate analysis	
	*P*	OR (95%CI)	*P*	OR (95%CI)
Gender				
Female		Reference		
Male	0.51	0.71 (0.26-1.94)		
Age (years)				
≤50		Reference		
50-60	0.404	0.59 (0.17-2.03)		
>60	**0.021**	0.24 (0.07-0.81)		
Family history of gastrointestinal cancer				
Absent		Reference		
Present	0.366	1.71 (0.53-5.50)		
Smoking				
Absent		Reference		
Present	0.622	1.25 (0.51-3.08)		
Alcoholism				
Absent		Reference		
Present	0.972	0.98 (0.34-2.81)		
FOBT				
Negative		Reference		
Positive	0.236	1.92 (0.65-5.64)		
Histologic type				
Differentiated		Reference		
Undifferentiated	**0.002**	4.47 (1.72-11.61)	**0.028**	3.12 (1.16-9.02)
CT-reported LN status				
Absent		Reference		
Present	**0.046**	3.09 (1.02-9.38)		
Tumor location				
Cardia/fundus		Reference		
Gastric body	0.258	2.60 (0.50-13.60)		
Antrum/pylorus	**0.035**	3.94 (1.10-14.08)		
Tumor size (cm)				
≤2		Reference		Reference
2-3	**0.002**	6.28 (1.99-19.76)	**0.008**	5.03 (1.57-17.80)
>3	**0.001**	8.70 (2.55-29.75)	**0.004**	6.59 (1.85-25.00)
Macroscopic type				
Protruded		Reference		
Superficial	0.767	1.37 (0.17-11.26)		
Excavated	**0.046**	8.80 (1.04-74.38)		
Ulceration				
Absent		Reference		Reference
Present	**0.009**	7.17 (1.63-31.59)	**0.038**	5.06 (1.33-33.20)
Atrophic/Intestinal metaplasia background				
Absent		Reference		
Present	0.901	1.06 (0.42-2.68)		
Spontaneous bleeding				
Absent		Reference		
Present	0.248	0.51 (0.17-1.59)		
The color of the lesion				
Red		Reference		
White	0.759	0.82 (0.23-2.93)		
Clear boundaries				
Absent		Reference		
Present	0.934	1.05 (0.34-3.28)		
ALB (g/L)	0.989	1.00 (0.90-1.11)		
Fib (g/L)	0.284	1.44 (0.74-2.82)		
LHD (mmol/L)	0.277	1.01 (0.99-1.02)		
WBC (10^9^/L)	0.533	0.92 (0.70-1.20)		
NE (10^9^/L)	0.588	0.92 (0.68-1.25)		
LY (10^9^/L)	0.736	0.88 (0.43-1.82)		
MO (10^9^/L)	0.704	1.78 (0.09-34.8)		
HGB (g/L)	0.671	1.00 (0.97-1.02)		
NLR	0.305	1.08 (0.93-1.25)		
MLR	0.730	1.58 (0.12-21.26)		

OR, Odds Ratio; CI, Confidence Interval; FOBT, fecal occult blood test; LN, lymph node; ALB, albumin; Fib, fibrinogen; LHD, lactate dehydrogenase; WBC, white blood cells; NE, neutrophils; LY, lymphocytes; MO, monocytes; HGB, hemoglobin; NLR, neutrophil-to-lymphocyte ratio; MLR, monocyte-to-lymphocyte ratio. Bold values indicate statistically significant results (P < 0.05).

### 3.3 Construction and evaluation of a nomogram for predicting lymph node metastasis

We developed a nomogram based on the three identified independent risk factors (**Figure 2B**). Each variable is assigned a score (ranging from 0 to 100) based on its value, with the total score (ranging from 0 to 280) indicating the probability of LNM. A ROC curve was constructed to evaluate the nomogram’s performance, yielding an AUC of 0.833 (95% CI: 0.757–0.909) (**Figure 2C**), demonstrating high predictive accuracy. The calibration curve (**Figure 2D**) indicated good agreement between predicted and observed outcomes. The decision curve (**Figure 2E**) analysis showed that the nomogram had favorable clinical benefits. Due to the lack of external validation in this study, we performed internal validation using the bootstrap method to assess the model’s predictive performance. After 1,000 bootstrap resamples, the model showed an accuracy of 0.896716, and the ROC curve was plotted (**Figure 2F**). The blue curve’s close proximity to the yellow curve indicated that the model’s performance is stable and reliable.

### 3.4 Identification and validation of lymph node metastasis-associated biomarkers

**Figure 3:**
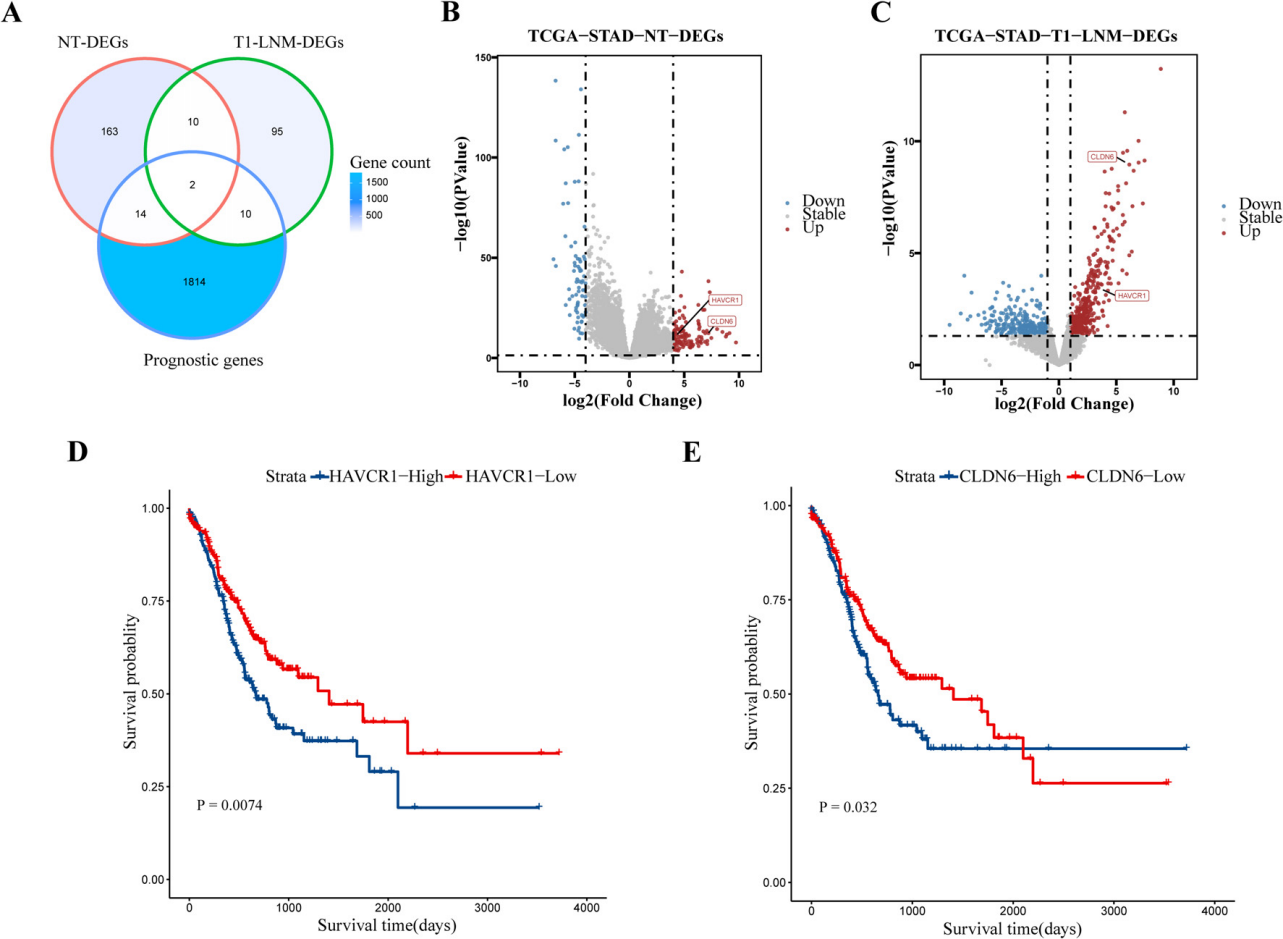
Identification and prognostic analysis of lymph node metastasis-associated biomarkers. **(A)** A Venn diagram shows the intersection of highly expressed differentially expressed genes in gastric cancer tissues, differentially expressed genes in early-stage (T1) gastric cancer tissues with lymph node metastasis, and prognostic genes, identifying two biomarkers. **(B, C)** Volcano plots display the high expression of these two biomarkers (*HAVCR1* and *CLDN6*)in gastric cancer tissues and early gastric cancer tissues with lymph node metastasis. Significant thresholds were set at *P*-value < 0.05 and |log2 Fold Change| > 4.0 and |log2 Fold Change| > 1.0, respectively. **(D)** Survival curve illustrating the prognostic value of *HAVCR1* in gastric cancer patients. **(E)** Survival curve illustrating the prognostic value of *CLDN6* in gastric cancer patients. *P* < 0.05 is considered to be statistically significant. LNM, lymph node metastasis; NT, Normal-vs-Tumor; DEGs, differentially expressed genes; TCGA, The Cancer Genome Atlas; STAD, stomach adenocarcinoma.

**Figure 4:**
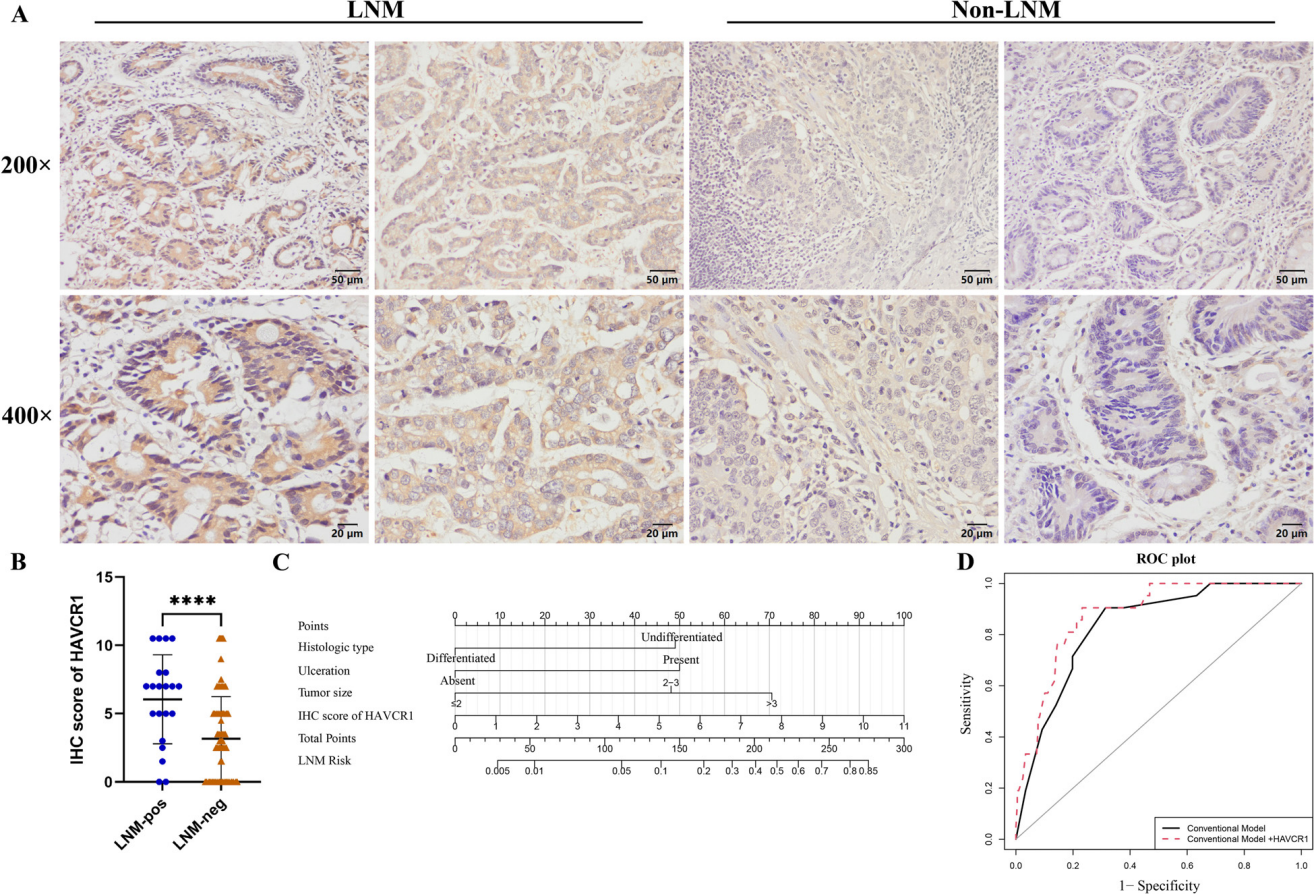
Representative images of *HAVCR1* immunohistochemical staining, statistical analysis, and evaluation of *HAVCR1*’s incremental impact on the conventional model. **(A)** Representative images of *HAVCR1* immunohistochemical staining in early gastric cancer tissues with and without lymph node metastasis. **(B)** Statistical analysis of *HAVCR1* expression in early gastric cancer samples with and without lymph node metastasis based on immunohistochemical scores. **(C)** The novel nomogram for the prediction of lymph node metastasis in early gastric cancer. The new model includes histologic type, presence of ulceration, tumor size, and IHC score of *HAVCR1*. **(D)** Receiver operating characteristic curves showing the incremental impact on predictive accuracy after incorporating HAVCR1 into the conventional model. **** *P* < 0.0001, *P* < 0.05 is considered to be statistically significant. LNM, lymph node metastasis; IHC, immunohistochemical; ROC, receiver operating characteristic.

To further enhance the predictive accuracy of our model, we identified biomarkers associated with LNM in early gastric cancer using data mining from the TCGA database. Through the intersection of differentially expressed genes (DEGs) that are highly expressed in gastric cancer tissues with those DEGs highly expressed in early-stage (T1) gastric cancer tissues exhibiting LNM, and with prognostic genes, we identified two biomarkers: *HAVCR1* and Claudin 6 (*CLDN6*) (**Figure 3A**). Volcano plots (**Figures 3B, C**) demonstrated that these biomarkers were significantly upregulated in EGC tissues with LNM. Survival curves (**Figures 3D, E**) revealed that high expression of *HAVCR1* and *CLDN6* correlated with poorer prognosis. IHC was subsequently performed to validate the protein expression levels of these biomarkers. Notably, preliminary experiments indicated that *HAVCR1* protein expression showed significant differences in metastatic gastric cancer tissues, leading to its selection for further validation as a LNM marker. IHC analysis of preoperative biopsy samples indicated that *HAVCR1* positive staining was primarily observed on the cell membrane and cytoplasm of tumor cells. **Figure 4A** illustrates the IHC staining patterns of *HAVCR1* in different specimens. We found that the IHC score for *HAVCR1* was significantly higher in EGC patients with LNM compared to those without LNM (**Figure 4B**).

### 3.5 Correlation of *HAVCR1* expression with clinicopathological features in early gastric cancer patients and evaluation of its incremental predictive value for lymph node metastasis

We further analyzed the correlation between *HAVCR1* expression levels and clinicopathological parameters in EGC patients’ gastric tissue samples (**Supplementary Table S1**), using a median IHC score of 3.5 as the cutoff for high and low expression. High *HAVCR1* expression was observed in 111 (48.7%) EGC patient samples and was significantly associated with LNM (*P* = 0.008), with no significant correlation with other clinicopathological factors. To evaluate the impact of *HAVCR1* on the predictive model, we incorporated its IHC score into the conventional model to predict LNM probability. The new model includes histologic type, presence of ulceration, tumor size, and IHC score of *HAVCR1*. We then developed a novel nomogram predictive model (**Figure 4C**). We found that adding *HAVCR1* resulted in an incremental effect on the AUC compared to the conventional model (AUC: conventional model + *HAVCR1*, 0.878 vs. conventional model, 0.833, *P* = 0.022) (**Figure 4D**). Additionally, it improved the model’s reclassification and discrimination abilities (NRI = 0.6749, *P* = 0.001; IDI = 0.0879, *P* = 0.015) (**Table 3**).

**Table 3:** The concordance statistic, net reclassification improvement, and integrated discrimination improvement values for predicting lymph node metastasis by immunohistochemical scores of *HAVCR1*.

Model	C-statistic Estimate (95% CI)	*P* value	NRI Estimate (95% CI)	*P* value	IDI Estimate (95% CI)	*P* value
**Conventional model**	0.833(0.757-0.909)	**0.022**	Reference	**0.001**	Reference	**0.015**
**Conventional model +IHC score of *HAVCR1***	0.878 (0.816-0.939)		0.6749 (0.267- 1.083)		0.0879 (0.017- 0.159)	

C-statistic, concordance statistic; NRI, net reclassification improvement; IDI, integrated discrimination improvement; CI, Confidence Interval. Bold values indicate statistically significant results (P < 0.05).

## 4 Discussion

Due to the proactive implementation of national upper gastrointestinal cancer screening programs and the widespread use of gastroscopy among high-risk populations, an increasing number of EGC cases are being detected and diagnosed promptly (28). For EGC patients with no risk of LNM, minimally invasive treatments such as EMR and ESD offer the advantage of preserving gastric function and maintaining quality of life (29). These procedures have emerged as viable alternatives to more invasive surgeries. LNM is considered a key prognostic factor in EGC (30). Since endoscopic resection cannot achieve perigastric lymph node dissection, surgical intervention is still required to achieve curative tumor resection in EGC patients with LNM (31). Therefore, accurately determining the status of LNM is a crucial factor in selecting the most appropriate treatment strategy for EGC (32).

In this study, the incidence of LNM among all EGC patients was 9.2%, which is consistent with or lower than the results reported in most previous studies (10, 33–35). This may be attributed to the relatively high proportion of T1a stage patients in our cohort. In our single-center study, we included a total of 26 preoperatively accessible variables covering various aspects such as pathology, imaging, endoscopy, blood tests, and clinical characteristics, as well as personal history, to comprehensively identify risk factors associated with LNM in EGC patients. We found that tumor size, histologic type, and the presence of ulceration were independent risk factors for LNM in these patients, which is consistent with previous studies (14, 36, 37). Studies have reported a significant association between tumor size and LNM (9). Our study similarly found that the larger the tumor, the higher the risk of lymph node metastasis, with this significance being notably greater than that of other risk factors. A multi-cohort study (13) identified varying degrees of differentiation in EGC as independent risk factors for LNM. Our study also demonstrated that patients with undifferentiated-type cancer have a significantly higher risk of LNM. This finding underscores the importance of accurate preoperative biopsy pathology reports. The presence of an ulcer is also a crucial risk factor that cannot be ignored in clinical practice. It plays a role in determining both the absolute and expanded indications for endoscopic resection (7).

Based on these risk factors, we constructed a predictive model presented in the form of a nomogram. The model demonstrated high clinical predictive performance, as evidenced by ROC curves, DCA, and calibration curves, and underwent internal validation. To further enhance the predictive capability of the model, we incorporated the immunohistochemical expression results of the biomarker *HAVCR1*. The inclusion of this biomarker improved the sensitivity, specificity, reclassification ability, and discrimination capacity of the final model in predicting LNM. The nomogram-based scoring system for predicting the risk of LNM will greatly benefit the selection of treatment strategies for patients with EGC. To the best of our knowledge, this is the first study to utilize transcriptomics to identify biomarkers for immunohistochemical validation in preoperative biopsy tissues, combined with preoperative clinicopathological factors, to establish a model for predicting the likelihood of LNM in EGC. Our predictive model is based on variables that are readily available preoperatively, enabling an initial assessment of LNM risk prior to surgery. It enhances clinically actionable therapeutic guidance for EGC patients following endoscopic examination and biopsy.

In addition to clinicopathological factors, molecular biomarkers are increasingly being utilized to predict LNM in gastric cancer patients (21, 22, 38–41). In our study, transcriptomic analysis revealed that the mRNA expression level of *HAVCR1* is higher in GC tissues compared to adjacent normal tissues, which is consistent with the findings of Liu et al. (42). Our study further demonstrates that *HAVCR1* is highly expressed in early gastric cancer patients with lymph node metastasis. The *HAVCR1* gene encodes a type I transmembrane glycoprotein (43), which is upregulated in various cancers, including esophageal cancer, lung adenocarcinoma, and gastric adenocarcinoma with different pathological characteristics, and its expression is associated with poor prognosis in these cancers (42, 44, 45). Studies have shown that the overexpression of *HAVCR1* can lead to the disruption of tight junctions between cells, which in turn facilitates cancer cell metastasis (46). Additionally, Xue et al. found that *HAVCR1* may promote the progression of gastric adenocarcinoma through the MEK/ERK pathway (47). Furthermore, other studies have demonstrated that *HAVCR1* plays a significant role in regulating and activating immune responses (48). Given these findings, *HAVCR1* holds potential as a target for tumor therapy. Our study found that *HAVCR1* is a sensitive biomarker for determining LNM in EGC, both at the mRNA and protein levels, and its IHC expression is independent of other clinicopathological factors.

This study has certain limitations. First, it is a single-center retrospective study with a relatively small sample size. Although the model’s internal validation showed good performance, external validation is lacking. We are committed to addressing this limitation in future research. Our goal is to collaborate with multiple institutions to collect larger and more diverse datasets, enabling external validation and further enhancing the generalizability of our model. Second, there is a selection bias as this study only included patients who underwent gastrectomy, excluding those who received minimally invasive treatments for EGC. Third, due to the fact that the patient enrollment period in this study spans over a decade, advancements in medical technology may have influenced the assessment of endoscopic lesions, pathological examination methods, intraoperative lymph node dissection, and the experience of endoscopists and pathologists, potentially affecting the accuracy of LNM prediction. Fourth, due to the long-time span of the study, some preoperative clinical factors, such as serum tumor markers, Helicobacter pylori antibody testing, and endoscopic ultrasound, were not included in the analysis. Fifth, not all patients had preoperative biopsy specimens tested, and some were diagnosed at other centers, leading to the use of final resection specimens for immunohistochemical validation. Finally, the mechanism by which *HAVCR1* is involved in LNM in EGC remains unclear and requires further experimental validation in future studies. Despite these limitations, the strength of our study lies in the incorporation of immunohistochemical expression of biomarkers into the conventional model development process. These factors are strictly available preoperatively, offering practical clinical utility in helping EGC patients select appropriate treatment methods.

## 5 Conclusion

In conclusion, our study identified the presence of ulcers during endoscopy, tumor size, and biopsy histologic type as independent risk factors for LNM in EGC. We developed a conventional nomogram model based on these factors. By incorporating the immunohistochemical expression of *HAVCR1*, a biomarker associated with LNM in EGC identified through transcriptomic analysis, we developed a new model based on the conventional model. The predictive performance of this new model, as assessed by the C-statistic, NRI, and IDI, showed significant improvement over the conventional model.

## Data Availability

TCGA-STAD datasets analyzed for this study can be found in the TCGA database (https://portal.gdc.cancer.gov/). The datasets used and/or analyzed during the current study are available from the corresponding author on reasonable request.
