# Supplementary material for: Combining biomarkers to construct a novel predictive model for predicting preoperative lymph node metastasis in early gastric cancer
Source: Front Oncol. 2025 May 8;15:1533889. doi: 10.3389/fonc.2025.1533889 (PMC12094995; doi:10.3389/fonc.2025.1533889)
Supplement: Supplementary file 1 [file Table1.docx]

| **Supplementary Table 1** Correlation between HAVCR1 expression and clinicopathological factors in early gastric cancer | | | | |
| --- | --- | --- | --- | --- |
| Variables | Overall (n= 228) | HAVCR1 expression | | p-value |
|  |  | Low(n= 117) | High(n= 111) |  |
| Gender |  |  |  | 0.245 |
| Female | 52 (22.8%) | 23 (19.7%) | 29 (26.1%) |  |
| Male | 176 (77.2%) | 94 (80.3%) | 82 (73.9%) |  |
| Age (years) |  |  |  | 0.329 |
| ≤50 | 25 (11.0%) | 15 (12.8%) | 10 (9.0%) |  |
| 50-60 | 62 (27.2%) | 35 (29.9%) | 27 (24.3%) |  |
| ＞60 | 141 (61.8%) | 67 (57.3%) | 74 (66.7%) |  |
| Family history of gastrointestinal cancer |  |  |  | 0.123 |
| Absent | 199 (87.3%) | 106 (90.6%) | 93 (83.8%) |  |
| Present | 29 (12.7%) | 11 (9.4%) | 18 (16.2%) |  |
| Smoking |  |  |  | 0.743 |
| Absent | 131 (57.5%) | 66 (56.4%) | 65 (58.6%) |  |
| Present | 97 (42.5%) | 51 (43.6%) | 46 (41.4%) |  |
| Alcoholism |  |  |  | 0.242 |
| Absent | 173 (75.9%) | 85 (72.6%) | 88 (79.3%) |  |
| Present | 55 (24.1%) | 32 (27.4%) | 23 (20.7%) |  |
| FOBT |  |  |  | 0.342 |
| Negative | 194 (85.1%) | 97 (82.9%) | 97 (87.4%) |  |
| Positive | 34 (14.9%) | 20 (17.1%) | 14 (12.6%) |  |
| Histologic type |  |  |  | 0.774 |
| Differentiated | 150 (65.8%) | 78 (66.7%) | 72 (64.9%) |  |
| Undifferentiated | 78 (34.2%) | 39 (33.3%) | 39 (35.1%) |  |
| Lymph node metastasis |  |  |  | **0.008** |
| Non-LMN | 207 (90.8%) | 112 (95.7%) | 95 (85.6%) |  |
| LMN | 21 (9.2%) | 5 (4.3%) | 16 (14.4%) |  |
| CT-reported LN status |  |  |  | 0.892 |
| Absent | 204 (89.5%) | 105 (89.7%) | 99 (89.2%) |  |
| Present | 24 (10.5%) | 12 (10.3%) | 12 (10.8%) |  |
| Tumor location |  |  |  | 0.450 |
| Cardia/fundus | 81 (35.5%) | 37 (31.6%) | 44 (39.6%) |  |
| Gastric body | 33 (14.5%) | 18 (15.4%) | 15 (13.5%) |  |
| Antrum/pylorus | 114 (50.0%) | 62 (53.0%) | 52 (46.8%) |  |
| Tumor size (cm) |  |  |  | 0.815 |
| ≤2 | 148 (64.9%) | 78 (66.7%) | 70 (63.1%) |  |
| 2-3 | 50 (21.9%) | 25 (21.4%) | 25 (22.5%) |  |
| >3 | 30 (13.2%) | 14 (12.0%) | 16 (14.4%) |  |
| Macroscopic type |  |  |  | 0.405 |
| Protruded | 23 (10.1%) | 9 (7.7%) | 14 (12.6%) |  |
| Superficial | 170 (74.6%) | 91 (77.8%) | 79 (71.2%) |  |
| Excavated | 35 (15.4%) | 17 (14.5%) | 18 (16.2%) |  |
| Ulceration |  |  |  | 0.204 |
| Absent | 91 (39.9%) | 42 (35.9%) | 49 (44.1%) |  |
| Present | 137 (60.1%) | 75 (64.1%) | 62 (55.9%) |  |
| Atrophic/Intestinal metaplasia background |  |  |  | 0.563 |
| Absent | 144 (63.2%) | 76 (65.0%) | 68 (61.3%) |  |
| Present | 84 (36.8%) | 41 (35.0%) | 43 (38.7%) |  |
| Spontaneous bleeding |  |  |  | 0.185 |
| Absent | 159 (69.7%) | 77 (65.8%) | 82 (73.9%) |  |
| Present | 69 (30.3%) | 40 (34.2%) | 29 (26.1%) |  |
| The color of the lesion |  |  |  | 0.051 |
| Red | 190 (83.3%) | 103 (88.0%) | 87 (78.4%) |  |
| White | 38 (16.7%) | 14 (12.0%) | 24 (21.6%) |  |
| Clear boundaries |  |  |  | 0.525 |
| Absent | 45 (19.7%) | 25 (21.4%) | 20 (18.0%) |  |
| Present | 183 (80.3%) | 92 (78.6%) | 91 (82.0%) |  |
| ALB (g/L) | 41.45 [39.08, 43.80] | 41.40 [38.90, 43.80] | 41.60 [39.75, 43.70] | 0.610 |
| Fib (g/L) | 2.92 [2.58, 3.25] | 2.92 [2.54, 3.24] | 2.94 [2.62, 3.26] | 0.751 |
| LHD (mmol/L) | 166.00 [148.00, 191.00] | 165.00 [147.00, 188.00] | 170.00 [149.00, 193.90] | 0.537 |
| WBC (10^9^ /L) | 5.55 [4.60, 6.75] | 5.52 [4.59, 6.80] | 5.58 [4.60, 6.72] | 0.920 |
| NE (10^9^ /L) | 3.17 [2.50, 4.16] | 3.20 [2.40, 4.20] | 3.14 [2.58, 4.10] | 0.938 |
| LY (10^9^ /L) | 1.60 [1.26, 2.07] | 1.60 [1.28, 2.07] | 1.60 [1.22, 2.07] | 0.833 |
| MO (10^9^ /L) | 0.38 [0.30, 0.50] | 0.40 [0.30, 0.50] | 0.38 [0.30, 0.49] | 0.953 |
| HGB (g/L) | 137.00 [125.00, 147.00] | 137.00 [125.00, 147.00] | 137.00 [125.00, 147.00] | 0.719 |
| NLR | 1.89 [1.38, 2.78] | 1.95 [1.38, 2.64] | 1.83 [1.41, 2.84] | 0.930 |
| MLR | 0.23 [0.17, 0.33] | 0.23 [0.17, 0.30] | 0.23 [0.17, 0.33] | 0.684 |
| FOBT fecal occult blood test, LNM lymph node metastasis, LN lymph node, ALB albumin, Fib fibrinogen, LHD lactate dehydrogenase, WBC white blood cells, NE neutrophils, LY lymphocytes, MO monocytes, HGB hemoglobin, NLR neutrophil-to-lymphocyte ratio, MLR monocyte-to-lymphocyte ratio | | | | |
